# Intranasally Administered EVs from hiPSC‐derived NSCs Alter the Transcriptomic Profile of Activated Microglia and Conserve Brain Function in an Alzheimer’s Model

**DOI:** 10.1002/alz.093071

**Published:** 2025-01-03

**Authors:** Leelavathi N Madhu, Maheedhar Kodali, Raghavendra Upadhya, Shama Rao, Bing Shuai, Yogish Somayaji, Sahithi Attaluri, Maha Kirmani, Shreyan Gupta, Nathaniel Maness, Xiaolan Rao, James Cai, Ashok K Shetty

**Affiliations:** ^1^ Institute for Regenerative Medicine, Department of Cell Biology and Genetics, School of Medicine, Texas A&M University Health Science Center, College Station, Texas, USA., College Station, TX USA; ^2^ Department of Veterinary Integrative Biosciences, Texas A&M College of Veterinary Medicine, College Station, Texas, USA., College Station, TX USA

## Abstract

**Background:**

Current treatments for Alzheimer’s disease (AD) lack disease‐modifying interventions. Hence, novel therapies capable of restraining AD progression and maintaining better brain function for extended periods after the initial diagnosis have great significance. Extracellular vesicles (EVs) from human induced pluripotent stem cell (hiPSC)‐derived neural stem cells (NSCs) are attractive in this context due to their robust antiinflammatory properties.

**Method:**

Three‐month‐old 5x familial AD (5xFAD) mice received IN administration of hiPSC‐NSC‐EVs (∼30 billion EVs) or the vehicle (weekly for two weeks). Seventy‐two hours later, a cohort of mice was euthanized, fresh microglia were isolated from the brain, and the expression of genes linked to DAM and NLRP3‐inflammasome signaling was examined via scRNA‐sequencing. Another cohort of 5xFAD mice was investigated for cognitive and mood function commencing one‐month post‐EV/Veh administration and euthanized two months post‐EV/Veh administration to assess the effects on long‐term progressive increases in neuroinflammation, amyloid‐beta plaques, and phosphorylated tau (p‐tau).

**Result:**

Microglia in all brain regions, including plaque‐associated microglia, internalized hiPSC‐NSC‐EVs entering the brain. Single‐cell RNA sequencing at 72 hours post‐EV administration revealed microglia with transcriptomic changes indicative of diminished activation. Multiple genes linked to disease‐associated microglia, NOD‐, LRR‐ and pyrin domain‐containing protein 3 (NLRP3)‐inflammasome and interferon‐1 signaling displayed reduced expression. Adding hiPSC‐NSC‐EVs to cultured human microglia challenged with amyloid‐beta oligomers also resulted in similar effects. Furthermore, the modulatory effects of hiPSC‐NSC‐EVs on microglia in the hippocampus persisted even two months post‐EV treatment, evidenced by reductions in microglial clusters and inflammasome complexes, concentrations of mediators, and end products of NLRP3 inflammasome activation, the expression of genes and proteins involved in p38/mitogen‐activated protein kinase hyperactivation, and proteins linked to activation of interferon‐1 signaling. The hippocampus also exhibited reduced amyloid‐beta plaques and p‐tau. Such modulatory effects of hiPSC‐NSC‐EVs also led to better cognitive and mood function.

**Conclusion:**

Early hiPSC‐NSC‐EV intervention in AD can maintain better brain function by reducing adverse neuroinflammatory signaling cascades, amyloid‐beta plaque load, and p‐tau.

Supported by a grant from the National Institute for Aging (1RF1AG074256‐01A1 to A.K.S.)

